# Aspirations and Worries: The Role of Parental Intrinsic Motivation in Establishing Oral Health Practices for Indigenous Children

**DOI:** 10.3390/ijerph182111695

**Published:** 2021-11-07

**Authors:** Brianna F. Poirier, Joanne Hedges, Lisa G. Smithers, Megan Moskos, Lisa M. Jamieson

**Affiliations:** 1 Australian Research Centre for Population Oral Health, Adelaide Dental School, University of Adelaide, Adelaide 5000, Australia; Joanne.hedges@adelaide.edu.au (J.H.); Lisa.jamieson@adelaide.edu.au (L.M.J.); 2 School of Public Health and the Robinson Research Institute, University of Adelaide, Adelaide 5000, Australia; lsmithers@uow.edu.au; 3 School of Health and Society, University of Wollongong, Wollongong 2522, Australia; 4 Future of Employment and Skills Research Centre, School of Economic and Public Policy, Faculty of the Professions, University of Adelaide, Adelaide 5000, Australia; Megan.moskos@adelaide.edu.au

**Keywords:** Indigenous peoples, oral health, dental caries, public health dentistry, motivational interviewing

## Abstract

Aboriginal and Torres Strait Islander (respectfully, subsequently referred to as Indigenous) children in Australia experience oral disease at a higher rate than non-Indigenous children. A history of colonisation, government-enforced assimilation, racism, and cultural annihilation has had profound impacts on Indigenous health, reflected in oral health inequities sustained by Indigenous communities. Motivational interviewing was one of four components utilised in this project, which aimed to identify factors related to the increased occurrence of early childhood caries in Indigenous children. This qualitative analysis represents motivational interviews with 226 participants and explores parents’ motivations for establishing oral health and nutrition practices for their children. Findings suggest that parental aspirations and worries underscored motivations to establish oral health and nutrition behaviours for children in this project. Within aspirations, parents desired for children to ‘keep their teeth’ and avoid false teeth, have a positive appearance, and preserve self-esteem. Parental worries related to child pain, negative appearance, sugar consumption, poor community oral health and rotten teeth. A discussion of findings results in the following recommendations: (1) consideration of the whole self, including mental health, in future oral health programming and research; (2) implementation of community-wide oral health programming, beyond parent-child dyads; and (3) prioritisation of community knowledge and traditions in oral health programming.

## 1. Introduction

Dentistry was originally established as a surgical specialty, with generations of dentists trained in highly invasive, operatively based treatments, grounded in biomedical theories of disease at the individual level [1,2,3]. The futility of this biomedical approach, which largely ignores social determinants of health, has been critiqued over the past century [4,5]. Not only has the surgically focused dental approach failed to generate significant individual or population benefits [2], it is also a considerable economic burden, even for high-income countries [1]. The necessity for a paradigm shift from therapeutic to prevention approaches has been acknowledged and is arguably an ongoing process [6]. Oral health, as a public health approach to dentistry, values prevention and attempts to tackle foundational causes of oral disease, particularly upstream determinants and structural drivers of inequity [1]. While oral health professionals are taught to provide education for all patients, provision of education and overt recommendations are seldom sufficient for sustained behaviour change [7,8]. Many prevention and disease management approaches still rely on patient cooperation and compliance with preventive strategies [9], which at best are challenging to implement, and at worse are ignored [6]. Oral health policies, such as water fluoridation, and clinical measures, such as topical fluoride application, have addressed biological domains of oral health at a population-level prevention effort [10,11]. However, employing behaviour change theories in oral disease management provides an opportunity for increased adherence to prevention strategies at an individual level [12].

Evidence-based behaviour change models, while critical for the success of oral health promotion programs, have only recently been employed in dentistry [11,13,14,15,16]. The basis of this approach is grounded in psychological theories that aim to change behaviour to maintain or strengthen oral health [12]. Behaviour change interventions and strong communication between parents and oral health practitioners have been shown to promote health decision making regarding Early Childhood Caries (ECC) risk-related behaviours [15]. Theories utilised in oral health have ranged from the Health Belief Model [17], Theory of Reasoned Action [18], concepts of self-efficacy from Social Cognitive Theory [19] to Stages of Change from the Transtheoretical Model [20,21], among others. Often, a shared goal of these approaches is increased oral health literacy and self-efficacy due to an abundance of evidence identifying low literacy as a risk factor for oral diseases [22]. Conversely, Motivational Interviewing (MI) is an approach that works to enhance intrinsic motivation for ambivalent or unmotivated individuals who do not consider behaviour change necessary, resist suggestions, have low adherence to health behaviours, or are unable to justify reasons for action [23]. In contrast to other oral health education approaches, MI is an empathetic and supportive method underpinned by the notion that knowledge is insufficient to elicit behaviour change and that intrinsic motivation increases the likelihood of behaviour change [24]. The traditional use of imparting knowledge and advice can bring about change in health-related knowledge but techniques such as MI have shown promise in promoting adaptive health behaviours and reducing maladaptive behaviours, particularly where motivation and ambivalence are barriers to change [25].

MI was originally developed to address substance use disorders in 1983 [26] but has since been expanded to target a range of health conditions, including oral health [27,28,29]. Empathetic listening is a defining feature of MI, which places importance on authentic understanding of a patient through practitioner listening, rather than informing [27]. MI ascertains that individuals know what is best for themselves and suggests that practitioners need to work individually with patients to determine the most effective strategies for behaviour change [30]. The goal of MI is to understand the need for behaviour change from an individual’s perspective, through principles of empathy, rolling with resistance, pointing out discrepancies, and supporting self-efficacy [27,31,32]. The technical hypothesis underpinning MI is an implicit causal chain, through what is known as “change talk,” where patients verbalise arguments for change; the relational hypothesis of MI is the client–counsellor relationship and the therapeutic skills of empathetic understanding [25,31]. There are a combination of relational and technical influences and a variety of pathways through which MI can facilitate behaviour change [25]. Technical techniques that can improve behaviour change through MI include those that elicit participant arguments for change, reduce arguments for not changing, explore values, and those which look to the future [32]. Relational techniques include reframing, shifting focus, emphasising autonomy, overshooting, and coming alongside [32]. MI creates an exploratory atmosphere for participants to articulate personal values, capacities, and motives for behaviour change; emphasising an individual’s personal motivation for change [24]. The recognition of misalignment between oral health values and poor oral health behaviours creates an internal force for clients that supports behaviour change [33]. For an individual to change, one must be confident in their abilities and believe that change is valuable, therefore employing interventions that bolster psychosocial strengths for parents can be effective in achieving optimal oral health for children [34,35,36,37]. Environments supportive of one’s autonomy, ideally established through MI, where motivation and encouragement are provided and personal choices are respected, foster intrinsic motivation. Intrinsic motivation is the most autonomous form of motivation because the desired behaviour is not contingent on external forces and is therefore more likely to be sustained, even throughout changing circumstances [38].

Aboriginal and/or Torres Strait Islander (respectfully, subsequently referred to as ‘Indigenous’] children in Australia experience significantly higher levels of ECC than non-Indigenous children both nationally and in South Australia, across all age groups [39,40]. Consequences of poor oral health during childhood impact pain, self-esteem, growth, development, quality of life, speech, education attainment, eating, concentration, and sleeping [41,42,43,44,45,46]. Despite the serious ramifications of ECC, this disease is preventable in nature and can be managed with limited sugar consumption, oral hygiene, fluoride exposure, and dental visits [46,47,48]. ECC is the strongest indicator for oral disease during adulthood [44,49]. Parent influence is instrumental in defining childhood oral health practices underscoring the importance of ECC prevention efforts focused on parent beliefs, attitudes, and self-efficacy within the family setting [46,50,51,52]. Importantly, MI parallels cultural values of Indigenous peoples, including oral traditions of storytelling and yarning [53], respects self-determination and is better able to yield a holistic and contextual understanding of a given issue [54,55]. MI has previously been used to elicit oral health behaviour change with Indigenous mothers and children [15,28,49,56], as well as with non-Indigenous mothers, reducing both occurrence and severity of child carious lesions [14,28,56,57,58]. Published evidence on MI in oral health has largely reported on the effectiveness of MI as a technique to reduce clinical measures of ECC rather than an exploration of participant-identified motivators that facilitate health promoting behaviours directly contributing to decreased ECC occurrence and prevalence. Investigation of parent-identified motivators will enhance understanding for oral health practitioners, policy makers and researchers of existing motivations and potential pathways which can be capitalised upon to further strengthen parental motivation for good oral health. The wider research project aimed to ascertain the impact of MI on parental oral health behaviours; this paper aims to explore intrinsic motivations identified by parents of Indigenous children during motivational interviews, which can be employed to enhance effectiveness of future oral health prevention efforts.

## 2. Materials and Methods

### 2.1. Design

The findings presented in this paper are derived from the MI component of a randomised controlled trial of an ECC intervention. This trial was designed and carried out in partnership with Indigenous communities and families across South Australia. At baseline, 448 women pregnant with an Indigenous child were enrolled and randomly assigned to control (delayed intervention) or intervention groups. The intervention had four components, (1) dental care provision during pregnancy; (2) fluoride varnish application for children; (3) anticipatory guidance; and (4) MI. The protocol [59], primary quantitative results [60] and cohort profile [61] have been published. Motivational interviews were conducted with the intervention group at baseline and when the child was 6-, 12-, and 18 months; parents in the delayed intervention group received MI when the child was 24-, 30-, and 36 months. The directives for each motivational interview were (1) dental care during pregnancy; (2) importance of non-cariogenic drinks and foods for children; (3) importance of fluoride for ECC prevention; and (4) child’s first dental appointment (Supplementary S1). At the end of each interview, parents completed a worksheet where they indicated their primary goal from the session, how they were going to achieve their goal, supportive individuals who they were going to share their goal with, and tactics to overcome challenges that could prevent them from achieving their goal. The study was conducted according to the guidelines of the Declaration of Helsinki and approved by the University of Adelaide Human Research Ethics Committee (H-057-2010) and the Aboriginal Health Council of South Australia (04-09-362).

### 2.2. Participants

This qualitative analysis employed purposive sampling of motivational interviews based on MI fidelity scores of trained staff who conducted interviews. Fidelity is the degree to which an intervention is executed as intended, through concepts of competence and adherence [62]. Fidelity of MI was assessed for this trial to ensure scientific rigour and sound methodological approach [63] due to the contingency of MI success on interventionist competency in eliciting self-motivating statements from parents [64]. Four staff were trained in MI and conducted interviews with parents with varying degrees of fidelity and success. The decision to only include interviews completed by the individual staff member with the highest fidelity score in this analysis was made because these interviews contained the richest data, were best able to answer the research question, and the interviews were comparable with one another. The staff with the highest fidelity score is a senior Indigenous researcher who facilitated the establishment of trusting relationships and employed colloquial language, which strengthened relationality.

### 2.3. Analysis

Reflexive thematic analysis embraces the subjective skills and unique experiences one brings to a project, acknowledging that these factors inescapably impact data interpretation and identification of themes [65]. The primary author is a non-Indigenous researcher from Canada, who spent significant time familiarising herself with the data and the context in which it was collected prior to analysis. Working with the same communities and Indigenous health workers who participated in this project enhanced local contextual and cultural understandings. The senior Indigenous researcher (JH) who conducted the interviews and the project’s primary investigator (LMJ) have extensive experience working with Indigenous communities and health services across South Australia, these relationships facilitated recruitment, retention, and engagement. Interviews were conducted in English, audio-recorded, and transcribed verbatim. Braun and Clarke’s reflexive thematic analysis framework guided the analytic process [65,66]. NVivo 12 software (QSR International Pty Ltd. Version 12.6.1, Doncaster, Australia) was used to facilitate the management and analysis of the qualitative data. Interviews were coded inductively, without a structured codebook to provide space for organic identification of themes, grounded in the data. The primary author continuously liaised with the researcher who conducted the interviews to ensure meaningful interpretation that reflected participant experiences. Upon completion of coding, all data points were reviewed, and similar codes were further explored and collated for an iterative thematic analysis process.

## 3. Results

Parents discussed several motivators for establishing oral health practices in their children, which generally related to either aspirations or worries. Findings presented below represent discussions with 226 parents of Indigenous children aged 6–36 months, from 357 interviews.

### 3.1. Aspirations

**Figure 1:**
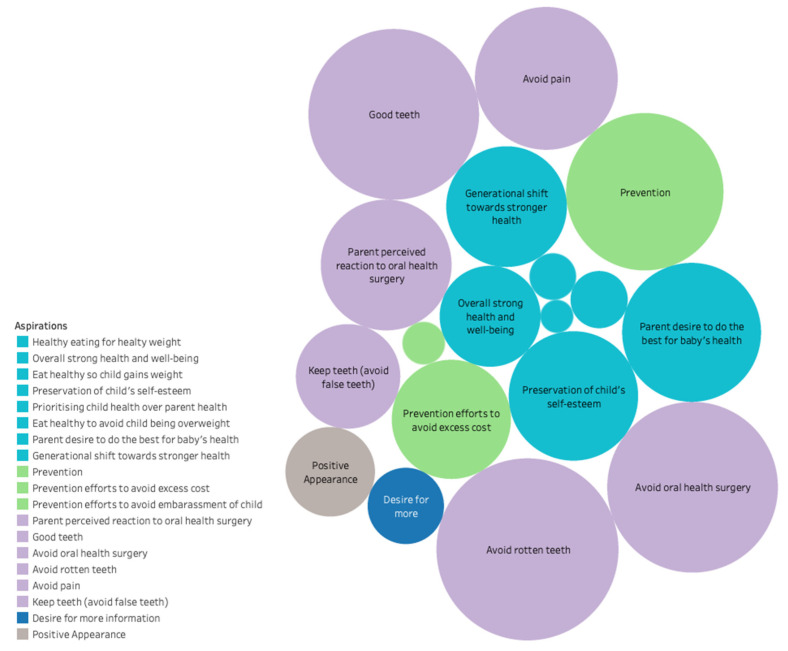
Parental aspirations contributing to intrinsic motivation. Note: The size of each bubble corresponds to the number of parents that discussed a given theme. Where more than one bubble has the same colour, the colour relates to general categories: purple with oral health, turquoise with general health, green with prevention, and grey with appearance.

Aspirations for parents broadly related to their child’s general wellbeing, as well as oral health (Figure 1). All parents desired to do what was best for their child’s health, regardless of their abilities, knowledge or circumstances, and often shared examples of prioritising their child’s health over their own health. Parents demonstrated pride when discussing their efforts for their child’s wellbeing as well as talking about their aspirations for their child’s future.

*“I’m very proud, they’ve got very good education, like, you know, they speak really well… I live for my kids and their education and health is my number one priority, I don’t give a crap about anyone else’s but these two are my driven force.”*

Aspirations for their child’s health included overall health and wellbeing, healthy eating for a healthy weight, generational shift towards stronger health and a preservation of child’s self-esteem. Parents were willing to take the necessary steps to ensure that their children had as few health problems as possible and many parents considered oral health to be an essential part of the child’s overall wellbeing: “*I want to be better for him because it’s his health, his health is his teeth and you know, [what’s] best for him, that’s all I want*.” Aspirations for healthy eating habits, in relation to healthy child weight was mentioned by some parents both in terms of gaining weight for underweight children as well as preventing the child from being overweight later in life. Aspirations for wellbeing also related to family longevity and strength, some parents discussed this in terms of the destructive impacts colonisation has had on their family line, and they had a strong desire to rebuild and maintain family health: “*Yes, well I think Aboriginals have been dated to be one of the first people that would die quicker and everything but so many years earlier than different nationalities so that’s a real battle… just to keep our race alive.*”

Where parents identified poor health or unhealthy habits in themselves or in relatives, parents were determined to initiate a generational shift towards stronger health for their children: “*My children are going to have a better life and upbringing than I did and that’s the most important thing for me.*” This also held true for parents with significantly older children who had witnessed the consequences of certain choices manifest as health difficulties; these parents were ready to take the necessary steps to prevent this from happening with their younger children. For some, a generational shift was a way for them to ensure their children “*[don’t] have to go through what I went through with my teeth.*” Many parents reflected on a lack of emphasis on oral health-related behaviours during their own childhood and highlighted a desire to prioritise oral health behaviours for their children: “*My Mum didn’t encourage me enough when I was a child to brush my teeth, so I try to encourage the kids more than I got encouraged when I was a child.”*

Parents emphasised the relationship between good oral health and mental wellbeing, aspiring to preserve self-esteem for their children. This association was grounded in personal experiences: “*To be honest, I’d like to be able to smile properly without wanting to hide all the time.*” Parents discussed the centrality of teeth to child confidence, self-image, and the ability to smile or laugh without embarrassment. Some parents also touched on the likelihood of teasing or bullying at school if their child had ‘rotten teeth.’ The desire to preserve self-esteem directly related to the very common aspiration of a positive physical appearance for children. Parents shared experiences of having difficulties securing employment or intimate relationships, which they partially or fully attributed to poor oral health. Parents did not want poor appearance, as related to oral health, to create preventable barriers for their children. Ultimately, oral health supported self-esteem and happiness for parents in this project and facilitated the best opportunities for children, which all parents inherently desired.

*“[If my kids have no fillings, they’ll] feel very pretty about themselves … pretty inside and outside and that’s something that every girl needs to feel. They need to feel secure about themselves and everything and if there’s a lot of Aboriginal girls out there with missing teeth, they don’t like it… not one little bit they don’t like it and not even the boys like it. Because we are very emotional people when it comes to our bodies and our hearts and our souls and everything you know, Aboriginals do care, in the end they do care, they might not show it but they do, yes.”*

In terms of child oral health, parents wanted clean teeth for their children, and to avoid oral health surgery, unnecessary pain, rotten teeth, and false teeth. Parents in this project expressed a desire for strong, white, or beautiful teeth for their children. For many, oral health was synonymous with general health and wellbeing; therefore, parents rarely distinguished the desire for health and the desire for healthy teeth as separate aspirations: *“Because I want him to have the best start with his health as well. Because I know once you have a lack of good hygiene with your teeth, that leads to other things. So that’s what I look ahead to when I think about him and his teeth.”*

Parents equally discussed the desire for healthy teeth and the desire to avoid rotten teeth. Many parents learned about the impacts of poor oral health from personal experiences: “*I had a lot of trouble with my teeth and I don’t want that for her*.” Parents also observed the impact of tooth decay from other people’s experiences: “*I don’t want his teeth to rot, it’s the last thing I want. I’ve seen it a lot and it’s not nice… poor kids, feel sorry for the kids because it’s got to be painful*.” Avoiding pain for children was one of the drivers for parents who aspired to avoid tooth decay. The desire to ‘keep teeth’ and avoid false teeth was discussed frequently, especially considering the very young age of children. Parents wanted children to have teeth that lasted for a long time and wanted to avoid false teeth before children were the age of 20 or 30 years, but for many, false teeth seemed to be an inevitable end point. Parents identified oral health surgery as scary, traumatic, and dangerous, especially for young children. There was a lot of hesitancy and discomfort for children to go under anaesthesia and many parents discussed the desire to prevent tooth decay, to avoid “unnecessary” surgery through oral health promoting behaviours at home: “*If you took the time to give them the right healthy diet, and look after their teeth, and got them to brush, and everything else, and if you looked after your own teeth, they wouldn’t [need surgery].”* In addition to the desire to avoid pain and other varying consequences associated with oral health surgery, many parents perceived negative child reactions to surgery as well as personal guilt or feelings of failure, which further fuelled the desire to avoid oral health surgery.

Parents highly valued prevention, identifying that the consequences of poor oral health, such as pain or surgery, are often “*not necessary*,” with many asserting that *“if [dental caries are] preventable, then I’ll do whatever I can, you know, to prevent that.”* Parents were motivated to take any necessary steps to strengthen prevention efforts for their children, including changing their own diets, modifying shopping habits, increasing personal oral health behaviours, and prioritising regular dental appointments for themselves and their children. For many, prevention was discussed as necessary for child wellbeing: “*We don’t want things that are preventable getting in the way of them doing whatever they want in their lives.*” Not only was prevention seen as critical for child oral health and general health, but parents also prioritised prevention in terms of avoiding excess health costs as well as avoiding embarrassment for their children as a result of poor oral health: “*The more I clean his teeth now the better they’ll be in the future and less trips to the dentist. Less money spent.”*

The majority of parents discussed a strong desire for more information regarding tangible steps they could take to further strengthen oral health efforts at home. Some parents talked about the dentist as a source of information and oral health education, while others felt constricted by appointment times. Desire for more information also extended to nutrition knowledge, while information pamphlets were helpful to some, others wanted a more hands-on experience such as help with grocery shopping.

### 3.2. Worries

**Figure 2:**
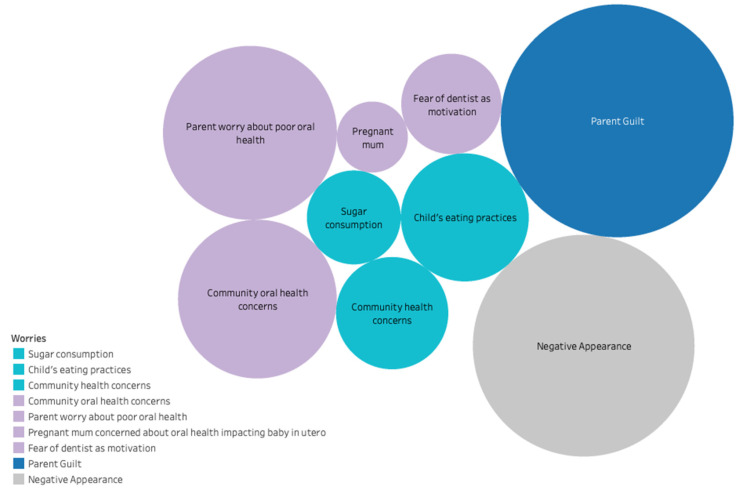
Parental worries contributing to intrinsic motivation. Note: The size of each bubble corresponds to the number of parents that discussed a given theme. Where more than one bubble has the same colour, the colour relates to general categories: purple with oral health, turquoise with general health, and grey with appearance.

Similar to aspirations, worries for parents generally related to child wellbeing and oral health as well as parental guilt, and negative appearances (Figure 2). Worries related to child health include child eating practices, community health concerns, as well as sugar consumption. Some parents discussed stress related to inadequate consumption of fruits and vegetables for their children, which motivated them to try new recipes or ways of incorporating fresh produce without children noticing, such as blended into pasta sauce. The child’s eating practices also related to concerns of high sugar consumption, and one mother discussed worries of high salt consumption. These worries motivated parents to change the accessibility of certain foods for children as a mechanism to limit exposure and prevent over-consumption.

*“I don’t really know what a lot of foods have in them as such. That’s why I kind of worry about a lot, because I know a lot of the foods that he eats, he really shouldn’t eat. Like, a lot of them I’ve cut down on. Like he hardly ever eats chocolate any more except for the chocolate milk... So I’m trying to keep as healthy as I can [with] beans, peas, corn and zucchinis and pumpkin, and he eats it all.”*

Concerns about widespread community health problems also contributed to parental worries for child wellbeing, especially regarding diabetes and obesity. Parents discussed high prevalence of diabetes in their families and communities: “*Diabetes is a big thing in communities. That’s another worry to look out [for]… [There’s] a high percent of kids being obese these days too.”* Awareness of the pervasiveness of chronic conditions motivated parents to make lifestyle changes for their children: “*Sugar runs very highly in my family, diabetes especially. I’ve cut down on bringing soft drinks in the house because [my partner] was really addicted to it and then [my child] got addicted to it as well. So now we don’t have it as much as we used to.*”

In terms of child oral health, parents worried about poor oral health, community oral health concerns, fear of the dentist, and pregnant mothers were concerned about the impacts of their oral health on the baby in utero. Parents worried about the impacts of tooth decay and gum problems for their children, which often related to a lack of correct oral health knowledge and a fear of doing the wrong thing for their children’s teeth. One parent also mentioned concern about the long wait time for her child’s dental appointment. Interviews with pregnant mothers revealed a lot of misinformation around dental visits during pregnancy, and a lack of understanding around the impact of maternal oral health on baby health. Many mothers were willing to take the necessary steps to limit the impacts of their oral health on baby health once made aware of this connection. Worries about poor oral health motivated parents to prioritise prevention efforts and establish oral health practices for their children: “*I reckon [the dentist] would say that [his teeth are] pretty good actually. He doesn’t eat lots of bad stuff, he mainly eats healthy stuff… I’ve always been paranoid about my kids, I don’t want my baby to be put under [general anaesthetic] because of something that I’ve fed him.*”

When discussing oral health surgery and tooth decay, many parents described the immense guilt they would feel if their child required surgery: “*I’d feel so guilty if she [needed oral health surgery]. Especially if it was something that could have been prevented like, by just caring for them.*” Some parents worried their children might fear the dentist when they are older and therefore prioritised regular dental checks to establish a good relationship with the dentist: “*His dad doesn’t want him to have teeth the way that he has them … which is very decayed right now because he’s too scared to go to the dentist. He doesn’t want [our son] to have that fear.*”

Community oral health concerns motivated parents in a similar way as general health concerns. Concerns related to the commonality of tooth decay and oral surgery in communities: “*You hardly never seen a black fellow smile mainly because they’ve got hardly no teeth in their heads or anything like that no more.*” Parents discussed limited attendance to dental appointments by relatives and neighbours: “*I mean most people I know and everything... I don’t think they take their kids to the dentist.*” Community oral health concerns and parent observations motivated a prioritisation of adherence to dental appointments for parents and establishment of oral health practices for children at a young age:

*“This is one thing that I worry about, that she’s going to have bad teeth because I’ve got bad teeth that run[s] in my family … When we went for a hospital appointment [we were told] one of the leading diseases is gum disease, for Aboriginal people, so I get really funny because that’s something that I was funny about growing up, was having bad teeth. So, yes, just to make sure that everything was okay, we decided we would take her [to the dentist] just to check that, yes, she’s where she’s supposed to be.”*

Negative appearance was the most frequently mentioned worry, with parents discussing the potential impact a negative physical appearance, as a result of poor oral health, would have on their children’s future. Some parents were worried about cosmetic implications of oral health, such as overcrowding of teeth or visible fillings, while others were motivated by the deeper implications of a negative self-image or shame associated with physical appearance of teeth. Negative physical appearance also related to parent embarrassment, many saw this as a visual indication of poor parenting: *“Yes, because I see other kids sometimes … and I just think it’s horrible … it would be embarrassing for me if I let my child have teeth like that.”* A few parents also discussed the ramifications of bad breath and confidence in talking to other people.

*“I just think overall, like personal appearance, you know, when they get older… I think part of me is my teeth. And if you have rotten teeth, you’re not really confident. You know what I mean? Like you don’t want to smile. You got stinky breath. You don’t want to breathe on people. You can’t eat certain things because your tooth breaks and falls out or, you know, all sorts of reasons, especially oral health.”*

## 4. Discussion

The aim of this paper was to explore factors motivating positive oral health and nutrition practices for parents of Indigenous children in South Australia. Findings suggest that aspirations and worries related to child oral health, child general wellbeing, child appearance, and community health trends underscored parental motivation. Within aspirations, parents desired a positive appearance for their children, to preserve child self-esteem and to keep their child’s teeth. Parental worries related to child pain, negative appearance, sugar consumption, community oral health concerns and rotten teeth. The findings highlight the relationship between parental motivations and the social and emotional impacts of oral health on children wellbeing.

Dental fear and anxieties have been associated with poor oral health for some time [67]. For parents in this project, dental fear was a motivator in terms of early exposure to dental services for children, in attempt to increase comfort and avoid experiences of fear. The relationships between poor oral health, quality of life, and self-esteem are evident in the literature [68,69]. In Australia, psychological distress has previously been associated with poor self-rated oral health for Indigenous peoples [70]. Experiences of shame in relation to poor oral health have been explored among Indigenous families in Western Australia, where participants described covering their mouths when laughing, staying home, and avoiding the dentist [71]. The stories shared by parents in this study are unique in that they provide narrative to parental perception and awareness of the potential ramifications of poor oral health on child mental wellbeing, particularly confidence and self-esteem, which often stemmed from personal experiences. Due to the recognised link between oral health and mental health, researchers and organisations in Australia have begun exploring an integrated approach to oral health and mental health [72,73,74]. One social worker in Western Australia highlighted the visible difference she observes in her clients, “*When people do actually get oral health managed and seen the difference that it makes is phenomenal… People are smiling. They look at themselves in the mirror more and then they take more pride in themselves and then they think of their opportunities*” [72]. Our findings acknowledge how parental experiences frame the importance of oral health prevention in strengthening social and emotional wellbeing for children.

Parents in this project were motivated to prioritise preventive oral health behaviours for their children due to worries of poor oral health, embarrassment of children. These motivators stemmed from personal experiences of oral health or observations of family and community oral health, similar to previous research with different Indigenous communities in Australia [75,76]. Previous research on child tooth brushing has also reported potential consequences of poor oral health, as a result of previous parent experiences, as motivating for parents [77]. Parents in this study mentioned prevention in terms of avoiding future oral-health related costs. Indigenous parents in Queensland have previously expressed concerns for the future of their children’s oral health due to personal experiences of inability to afford corrective treatment for oral health problems and tooth loss [75]. The importance of prevention to parents in this study contrasts previous reports of low prevention efforts among Indigenous peoples in Australia [49,78]. It is plausible that various barriers to accessing preventive services, such as cost, family responsibilities, waiting times, and distance [79,80], prevent parents from accessing oral health care despite the strong desire to take preventive steps exemplified by parents in this study. Future research should explore mechanisms to increase availability and accessibility of prevention services for Indigenous families and communities that aligns with Indigenous Australian values and aspirations. The desire for a generational shift towards stronger health for parents in this study related to findings of the Canadian extension of this trial, wherein grandmothers and local health knowledge keepers in Manitoba helped facilitate a similar shift by utilising culturally based childrearing practices for child oral health. Some of the practices employed and explored included traditional medicines in oral health, feeding children traditional foods from a young age, and the role of swaddling in healthy deciduous teeth development [81]. The importance of culture and intergenerational relations to parents in this project, parallels the importance of including community knowledge and traditions in oral health programming [81,82,83].

Discussions around community health and oral health concerns highlight parental awareness of community health trends, which have been extensively documented in the literature [39,40]. This finding is unique in that community health was not a discussion topic for interviews and multiple parents framed their child’s experience of oral health in the broader community context. Potentially, oral health programs and public health campaigns have contributed to increased parent awareness of community health. Observations of community trends motivated parents to establish oral health practices for their children. Children in this study were aged 3 years or younger and parents regularly discussed a desire to keep children’s teeth and avoid tooth loss. This finding alludes to the commonality of tooth loss and is similar to concerns expressed by Indigenous parents in Queensland who identified tooth loss as a common experience [75]. Indigenous parents of young children in Western Australia have also conveyed concern for the state of children’s oral health, “*You see a lot little kids who have rotten teeth*” [71]. Participants from a rural community in Queensland described community suffering due to oral disease as both frequent and ongoing [76]. Fatalistic mindset regarding tooth loss and normalcy of dental extraction in Indigenous populations has been noted both in Australia and New Zealand [75,84]. A linear relationship between the number of missing teeth and annual income exists in Australia, and globally [85,86,87]. Despite the range of communities involved in this project, there was a consensus of community health concerns across the entire state. However, in contrast to previous research, poor oral health was not described as inevitable by parents in this project, although it was a major concern, parents remained hopeful and motivated that establishing oral health practices could prevent poor outcomes for their children.

### Strengths and Limitations

MI enabled the identification of intrinsic motivators for parents in this study and the clinical measure of dmft was significantly reduced for children in the intervention group in comparison to the delayed intervention group [60]. While MI is typically employed to fulfil behaviour change objectives, thematic analysis of interviews has provided a unique opportunity to identify parent motivations for establishing oral health and nutrition practices and added to the limited qualitative research on Indigenous oral health in Australia. Endeavours to improve oral health for Indigenous children need to be culturally appropriate, non-judgmental, and informative [83]. For this project, MI fulfilled those needs by respecting oral traditions of yarning and facilitating active parent engagement. The number of interviews and families, as well as the timing of interviews from 6 to 36 months of child age, make it unlikely that any significant motivators were missed. The importance of the relational style and behaviour of the interviewer is central to both MI as well as Indigenous research. The relationship between the interviewer and participants is critical for garnering honest and open conversations. Therefore, it is unlikely that interviews would be reproducible by researchers without cultural sensitivity and the desire or ability to develop trusting relationships with participants. The time and cost of intensive MI training and fidelity assessment is a limitation of this approach. Due to baseline recruitment during pregnancy, the majority of conversations included in this analysis are with mothers, despite the notable importance of fathers, families, and communities in developing and maintaining child oral health.

## 5. Conclusions

Generally, aspirations and worries related to prevention, child health, child oral health, community health and child appearance for parents in this project. Findings highlight the relationship between parental motivation and the emotional and mental impacts of oral health on children. The influence of community health on parent understanding of child health in this project underscores the importance of community-level interventions in future programming. Oral health interventions typically focus on family, school, or clinical settings; co-designing programs held on Country that incorporate all community members, from young children to Elders, has the potential to not only strengthen community oral health knowledge but also oral health status. Oral health professionals, policy makers, and researchers are encouraged to utilise the motivators explored here to centre Indigenous voices and understandings of oral health in future work. Our recommendations from these findings include: (1) consideration of the whole self, including mental health, in future oral health programming and research; (2) implementation of community-wide oral health programming, beyond parent–child dyads; and (3) prioritisation of community knowledge and traditions in oral health programming.

## Data Availability

The data presented in this study are available upon reasonable request from the corresponding author. The data are not publicly available due to conditions of ethics approval.

## Supplementary Materials

The following are available online at https://www.mdpi.com/article/10.3390/ijerph182111695/s1, S1: Motivational interview prompts.
