# Supplementary material for: Aspirations and Worries: The Role of Parental Intrinsic Motivation in Establishing Oral Health Practices for Indigenous Children
Source: Int J Environ Res Public Health. 2021 Nov 7;18(21):11695. doi: 10.3390/ijerph182111695 (PMC8582669; doi:10.3390/ijerph182111695)
Supplement: Supplementary file 1 [file ijerph-18-11695-s001.zip › ijerph-1430669-supplementary.pdf]

S1– Motivational Interview Guides

<u>MI: 1</u>

Intro

1. Scope of study / spirit of MI
2. Timeline / info sheet
3. Consent / voucher & goodies / contact details

Discussion

1. Find out what she knows
    - Is everything going okay with your teeth?
    - Can you tell me what you think about oral health?

2. Cards: Agenda setting
   There are a number of things we can talk about today. These are some of the ways people work towards oral health. Some of these you might have heard of/are already doing; others might be new ideas for you. When you look at these cards, what would you like to find out more about today?
    - Take yourself to the dentist
        - – Dental treatment safe & important during pregnancy (pre-term, enamel, birth weight).
        - – How bugs in mouth can pass to baby
    - Brushing daily stops germs
        - – Soft toothbrush, fluoride toothpaste, evening better than morning, spit (don’t rinse)
        - – Brush bleeding gums gently to heal
    - Take care of your teeth
        - – Children learn by watching you, teaching values. Brushing teeth, regular trips to dentist, how decay spreads to babies.
    - Fresh food is best
        - – Bush tucker & veg low in sugar, germs + sugar=cavities, starchy foods are sugary
        - – Fruit, cheese, yogurt are good snacks. Drink tap water when thirsty. Dilute juices with water.

3. Ask-Provide-Ask
    - I’m happy to share ideas with you... what have you already thought of?
    - Some mothers do it this way... what do you make of that?
    - Can you think of other times when this happened, but you got through it?
    - From what you’re saying, it sounds like ___ may be an issue. What ahve you heard about ___?
    - There are some great ideas about the ways women look after their teeth during pregnancy... would it be okay to talk about some of these?

4. Rulers: Importance and Confidence
    - On a scale of 0 -10
    - High
        - What makes change important to you?
        - What does it mean to be a __?

- Low
    - How come a 2 and not a 1?
    - What would it take to bump you up a few notches?
    - What would help you feel more confident to make this change?

5. Commitment language
    - What do you think the first step might be to reach your goal?
    - Given that you're here... where would you like to go now?

6. Change Plan

7. Referral

<u>MI: 2</u>

*denotes activity

Intro
1. Scope of study / spirit of MI
2. Timeline / info sheet
3. Consent / voucher & goodies / contact details

Discussion
1. Establish rapport.
    - How are you and baby going? (e.g. family, birth, feeding)

2. Ask for change talk.
    - What are your goals for baby's teeth? What concerns might you have?
    - Why do you think it's important for your baby's teeth to come in healthy and strong?

3. Listening / sharing information.
   (Find out what she knows about looking after baby's teeth. → Discuss bugs/time/sugar in MI-consistent way.)

   Listening.
    - Can you tell me your thoughts about looking after your baby's oral health?
    - How is everything going with baby's teeth? (e.g. teething, sleeping/soothing)
        i. What are some of the things you're doing as _______ (e.g. baby's teeth come through; baby gets cranky)?

   Sharing information. (Ask – Provide – Ask / listen, reflect, summarize / encourage change talk)
   →From what you're saying, it sounds like ______ may be an issue. Is it alright if I share some information with you? We've learned that there are 3 important things which put holes in our teeth:

   BUGS

Babies aren't born with bugs in their mouth. Bugs come from people (e.g. through sharing food, cleaning dummy in mouth, kissing) and sugar (which combines with bugs to form acid, dissolving the teeth).

Fizzy dissolving teeth
When we eat food or drink, the bugs in our mouths break down the food and drink into ACID. The acid then starts to dissolve the healthy tooth. Bugs breakdown food/drink to make acid, and the acid makes holes in baby's teeth.

– Discuss. (Wiping away bugs with cloth.)

TIME

- The more frequently we eat or drink things that contain sugar, the more we dissolve our teeth. Which do you think is better: sipping your can of fizzy over cleaning the house, or drinking a can at dinner? It's better to finish your sweet drink quickly than having it over a long time.
- One of the main causes of children getting decay is going to sleep with a bottle of something other than water, let's say milk (which contains sugar). That sugar is getting eaten by the bugs to make acid. The acid is dissolving baby's teeth for the 8 hours they are sleeping.
  – Discuss. (Drinks at sleeping time do the most damage, water ok.)

SUGAR

- Sugar combines with bugs to form acid. Cariogenic substances; milk, formula, juice, flavoured milk, cordial, fizzy, lollies, high sugar content puree fruit, tinned fruit.

Ranking sugary drinks
Sugar in baby food Show where to find sugar on the label. A good amount would be under 5g.

– Discuss. (Breast milk and formula are best. Only milk or water should go in baby's bottle. Try not to give baby food with tooth much sugar.)

4. Use bugs/time/sugar cards to discuss issues relevant to mum. (Ask – Provide – Ask.) She can pick topics, and/or you could discuss these: "What do you know about _____ for baby's teeth?"
   - Cleaning
     – Wipe baby's gums and teeth with damp cloth, morning and night
     – Around 10 months, brush baby's teeth with toothpaste morning and night
     – Play with a toothbrush to encourage use
     – Less bugs = less decay
   - Healthy diet
     – Minimize sugar intake (e.g. juice, fizzy drinks, cordial, flavoured milk, honey, jam)
     – Breast milk is best
     – Discuss healthy foods/bush tucker
   - Teething
     – Give baby damp wash cloth, frozen ring, or cold spoon to suck on
     – See doctor about baby's pain relief

5. Rulers: Importance and Confidence

- On a scale of 0 -10
- High
    - What makes change important to you?
    - What does it mean to be a __?
- Low
    - How come a 2 and not a 1?
    - What would it take to bump you up a few notches?
    - What would help you feel more confident to make this change?

6. Commitment language
    - What do you think the first step might be to make sure baby has the best possible start for healthy, strong teeth?
    - Given that you're here... where would you like to go now?

7. Change Plan
    - What kinds of challenges do you see this plan?

<u>MI: 3</u> (12 months)
Goal: Fluoride varnish

Intro

Scope of study / spirit of MI. Timeline / changes to info sheet / fluoride questions. Audio consent / goody bag / contact details

Discussion

1. Intro
    - How is everything going with your baby's teeth?
    - The focus of our meeting today is on keeping baby's teeth healthy, especially with the help of fluoride. Can you tell me what you know about fluoride?
    - Ask for change talk:
        - What are your goals? What concerns do you have about fluoride varnish?
        - Why do you think it's important for baby's teeth to come in strong and healthy?

2. Agenda setting
   There are a number of things we can talk about today. These are some of the ways we can look after babys' oral health. Some of these you might have heard of/are already doing; others might be new ideas for you. When you look at these cards, what would you like to find out more about today?
    - Fluoride varnish
      <u>What</u>:
        - Fluoride is a natural mineral found throughout the earth's crust and widely distributed in nature.
        - Fluoride varnish is a protective coating which is painted on the teeth to stop cavities forming and to slow down cavities which may have started. The varnish last for 6 months. So your child should receive the fluoride varnish every 6 months for the next 2 years.

– Has a good taste. It's a sticky gel and stays on teeth for a long time, soaking deep into the tooth to make it very strong.
– It is safe and has been used all over the world.

How:
– Fluoride is in tap water, toothpaste, some foods, and also comes in a high concentration called fluoride varnish. This is the application of fluoride directly onto the tooth. It helps to rebuild damage produced by acids. FV has over 48 times the amount of fluoride as child toothpaste.
– Fluoride concentrates in the growing bones and developing teeth of children, helping to harden the enamel on baby and adult teeth before they emerge.
– Fluoride helps because, when teeth are growing, it mixes with tooth enamel (the hard coating on your teeth), that prevents tooth decay, or cavities. But fluoride can help even after your teeth are formed. It works with saliva to protect tooth enamel from plaque and sugars.
– Also helps even when the cavities have started

Why:
– This is a problem affecting Aboriginal kids in particular. Important to make change for Aboriginal health.
– Some people believe that fluoride causes cancer, but this not true and has never been proven. They also think that fluoride is bad for you because it is given to everyone, and they would rather individuals get to decide. But fluoride in water is actually the most effect public health initiative that's ever happened, anywhere in the world. What's really great about it is that the people with the most decay end up getting the biggest benefit from fluoride varnish—so it reduces inequalities. Before fluoride was put in our water in the 1950s, most people had to have all of their teeth pulled out.

When: Within the first 12 months, baby gets their top 4 and bottom 4 teeth—so this is the best time for fluoride varnish to work. We want to apply it today and also in 6 months, when baby is about 18 months old.

Who: Dental professional. In our study, staff are "dental professionals." You can't buy Duraphat at the Chemist or supermarket.

- No bottle feeding at night
  – The main cause of babies getting holes in their teeth is going to bed with a bottle of something other than water (like milk, which contains sugar). The sugar from the milk gets eaten by the bugs on our teeth to make acid. The acid dissolves baby's teeth for the 8 hours they are sleeping.
  – Drinks at sleeping time do the most damage. Water is okay.

- Brushing baby's teeth
  – Now is the time to start brushing baby's teeth, morning and night
  – Play with toothbrush to encourage use
  – Brushing off bugs from teeth prevents holes. Less bugs = less decay
  – Do not use toothpaste until 18 months

- Healthy diet for baby
    – Minimize sugar intake (e.g. juice, fizzy drinks, cordial, flavoured milk, honey, jam). A good amount from canned food is under 5g.
    – Breast milk is best
    – Bush tucker & vegetables low in sugar, germs + sugar = decay, starchy foods are sugary too
    – Drink tap water when thirsty. Dilute juices with water.

<u>Ask-Provide-Ask</u>

- I'm happy to share ideas with you... what have you already thought of?
- Some mothers do it this way... what do you make of that?
- Can you think of other times when this happened, but you got through it?
- From what you're saying, it sounds like ___ may be an issue. What have you heard about ___?
- There are some great ideas about the ways mums look after their baby's teeth... would it be okay to talk about some of these?

3. Rulers: Importance and Confidence

4. Commitment language
    - What do you think the first step might be to make sure your baby has the best possible start for healthy, strong teeth?
    - Given that you're here... where would you like to go now?

5. Change Plan

    My plan is to:
    It's important to me because:
    A step I can take is:
    Someone who can help is:
    If things don't go well, I can:

    - What kinds of challenges do you see to your plan?
    - What kinds of challenges do you see for continuing with fluoride varnish through this project? How about meeting us in 6 months to put more varnish on baby's teeth?

6. After applying FV, let mum know:
    1. No food or drink for at least 30 minutes
    2. No brushing or cleaning teeth tonight
    3. Teeth will have a yellow tint from the fluoride but will come off when brushed in the morning.
    4. I'll be back in 6 months for another application.

<u>MI: 4</u> (18 months)

Goal: schedule baby's dental check (+ apply FV)

Intro

1. Scope of study / spirit of MI

2. Timeline / changes to info sheet / fluoride questions
3. Audio consent / goody bag / contact details

Discussion

1. Intro
   - How is everything going with your baby's teeth?
   - The focus of our meeting today is on taking baby for her first dental check. Can you tell me what you think about taking baby to the dentist?
   - Ask for change talk around taking baby to the dentist:
     - – Can you tell me why you think it's important for you to have baby's teeth to come in strong and healthy?
     - – What are your goals for taking baby to the dentist? What concerns do you have about taking her?

2. Agenda setting
   There are a number of things we can talk about today concerning taking baby to the dentist. We can't know until we get there, but these 4 cards show some of the things that might happen when you take your baby to the dentist. When you look at these cards, what would you like to find out more about today?
   - Healthy check up / healthy teeth
     - – Things go well: baby gets comfortable with going to the dentist, starts a healthy habit from a young age which they can carry into adulthood, dentist will look at her teeth, clean them, etc.
     - – Diet, brushing, no bottles at night
   - Family support
     - – When the clinic calls, you can ask to bring your family with you. It could be a new family tradition, supporting each other by doing it together and maybe even holding each other accountable back at home too, to brush, eat healthy foods, etc. How can you make things better for your family now than what you've seen others do in the past? What kinds of changes do you want to make?
   - Treatment (crowns under GA)
     - – After the front teeth decay, the baby can't have crowns (which only work on molars).
     - – Crown is placed on tooth to keep it strong until it falls out naturally.
     - – There is a possibility that baby will need treatment for holes in her teeth. This could mean having crowns put in, or maybe even having teeth pulled out under a GA. At this age, the baby will need to go to a dentist/hospital for care. General Anaesthesia (GA) refers to being "put to sleep". During GA, the baby is unconscious. First they give you the baby gas to calm you down, then they numb the baby's arm with a spray before putting a needle in. This puts the baby to sleep.
       The baby can't drink or eat for 6 hours before the procedure (otherwise, vomiting is possible and this would be very dangerous during GA).
     - – Even though it's difficult, this is a really positive thing to do.

3. Ask-Provide-Ask / Ask for change talk
   - Can you think of other times when something difficult or scary happened, but you got through it?

- From what you're saying, it sounds like ___ may be an issue. What have you heard about ___?
- What are your goals? What concerns do you have?
- Why do you think it's important for baby's teeth to come in strong and healthy?

4. Rulers: Importance and Confidence
    - On a scale of 0 -10
    - High
        - What makes change important to you?
        - What does it mean to be a __?
    - Low
        - How come a 2 and not a 1?
        - What would it take to bump you up a few notches?
        - What would help you feel more confident to make this change?

5. Commitment language / plan:

    - What do you think the first step might be to make sure you can take baby to the dentist?
    - What kinds of challenges do you see to your plan?
    - What kinds of challenges do you see for continuing with fluoride varnish through this project? How about meeting us in 6 months to put more varnish on baby's teeth?

    My plan is to:
    It's important to me because:
    A step I can take is:
    Someone who can help is:
    If things don't go well, I can:
